# Disseminated rhodococcus equi infection in HIV infection despite highly active antiretroviral therapy

**DOI:** 10.1186/1471-2334-11-343

**Published:** 2011-12-14

**Authors:** Francesca Ferretti, Antonio Boschini, Cristiana Iabichino, Simonetta Gerevini, Paola De Nardi, Monica Guffanti, Giuseppe Balconi, Adriano Lazzarin, Paola Cinque

**Affiliations:** 1 Department of Infectious Diseases, San Raffaele Scientific Institute, Milano, Italy; 2 San Patrignano Medical Center, Rimini, Italy; 3 Department of Radiology, San Raffaele Scientific Institute, Milano, Italy; 4 Head and Neck Department, San Raffaele Scientific Institute, Milano, Italy; 5 Department of Surgery, San Raffaele Scientific Institute, Milano, Italy

## Abstract

**Background:**

*Rhodococcus equi *(R.equi) is an acid fast, GRAM + coccobacillus, which is widespread in the soil and causes pulmonary and extrapulmonary infections in immunocompromised people. In the context of HIV infection, *R.equi *infection (rhodococcosis) is regarded as an opportunistic disease, and its outcome is influenced by highly active antiretroviral therapy (HAART).

**Case presentation:**

We report two cases of HIV-related rhodococcosis that disseminated despite suppressive HAART and anti-rhodococcal treatment; in both cases there was no immunological recovery, with CD4+ cells count below 200/μL. In the first case, pulmonary rhodococcosis presented 6 months after initiation of HAART, and was followed by an extracerebral intracranial and a cerebral rhodococcal abscess 1 and 8 months, respectively, after onset of pulmonary infection. The second case was characterized by a protracted course with spread of infection to various organs, including subcutaneous tissue, skin, colon and other intra-abdominal tissues, and central nervous system; the spread started 4 years after clinical resolution of a first pulmonary manifestation and progressed over a period of 2 years.

**Conclusions:**

Our report highlights the importance of an effective immune recovery, despite fully suppressive HAART, along with anti-rhodococcal therapy, in order to clear rhodococcal infection.

## Background

*Rhodococcus equi *(R.equi) is an acid fast, GRAM + coccobacillus, which was first isolated from suppurative pulmonary lesions in foals [1]. The first human case of R.equi infection (rhodococcosis) was reported in 1967 in an immunocompromised patient with pneumonia [2] and its frequency has increased significantly during the last 20 years [3-5], especially in immunocompromised patients, such as transplant recipients and HIV-infected patients [6,7]. Rhodococcosis is a rare infection, the exact prevalence of which is not known. Until now, more than 200 cases have been reported worldwide [4,6].

In the majority of the cases, *R. equi *is acquired by inhalation or aerosols coming from the stool of infected foals. Excavated pneumonia is the most frequent clinical manifestation [8], although spreading of the infection to other organs is common, particularly in the immunocompromised subjects [9-15]. The diagnosis relies on radiological examinations [16], isolation of *R. equi *in blood, sputum and other body fluids [17], and histological examination of tissue samples, which may reveal typical necrotizing granulomatous lesions, also termed as malakoplakia [18].

There is no standard treatment for rhodococcosis and it usually consists of a combination of at least two antibiotics to which the agent is susceptible. These include macrolides, rifampin, floroquinolones, aminoglycosides, glycopeptides and carbapenems, although newer drugs, such as tygecicline and linezolid have also successfully been used [19-21]. The choice should be based on the results of antibiogram and drugs be given intravenously for at least 2 weeks, followed by prolonged oral suppressive antibiotic treatment [4]. Surgical drainage of abscesses or cavitary lesions may also be required [9]. Despite treatment, the outcome of rhodococcosis is poor in immunocompromised patients, with the highest mortality (50-60%) in HIV infection. The use of highly active antiretroviral therapy (HAART), however, has dramatically changed the prognosis in HIV-infected patients, with reported survival rates of virtually 100% [9]. The cellular immunity, in particular Th1 response, appears indeed to play a prominent role in the containment of *R. equi *infection [22].

We here report two cases of *R. equi *pneumonia in HIV-infected patients that disseminated despite virologically suppressive HAART, without CD4+ cell counts increase above 200/μL. These cases highlight the importance of an effective immune recovery induced by HAART, along with appropriate antibiotic therapy, in order to clear rhodococcal infection. In addition, they illustrate the wide spectrum of clinical manifestations caused by *R. equi *and the potential of non conventional radiological approaches, such as nuclear techniques, in the diagnostic work-up and follow-up of *R. equi *lesions.

## Case Presentation

### Case report 1

In April 2002 a 49 year-old HIV-infected woman was admitted to hospital for persistent fever above 38°C and cough (Table 1). She had started HAART with didanosine, lamivudine and indinavir in October 2001,when her CD4+ cells count was 118/μL, and 2 months later had developed brain and brain stem vasculitis-like contrast-enhancing, white matter lesions, consistent with immune reconstitution central nervous system (CNS) manifestations, for which she did not receive any treatment.

**Table 1:** Summary of main clinical, laboratory and imaging findings from patient 1

Onset of symptoms (date)	Presenting symptoms	Site of R. equi infection (diagnostic technique)	CD 4 + cells, HIV-1 RNA (VL)	HAART	Treatment	Duration
April 2002	fever, cough	lung (culture of sputum, chest X-ray). disseminated infection (blood culture)	123 cells/μl, VL < 50 copies/ml	didanosine, lamivudine, indinavir	induction (i.v.): vancomycin 500 mg q6h imipenem 500 mg q6h ceftriaxone 2 g qd ciprofloxacin 400 mg bid maintanance (p.o.): ciprofloxacin 500 mg bid	induction: 8 weeks maintanance:15 weeks

May 2002	seizures	head (brain MRI, culture of surgically removed brain abscess)		didanosine, lamivudine,indinavir	surgery, antibiotic therapy (see above)	

December 2002	anisocoria, right hemiparesis, hypoaesthesia, hypoallaesthesia, dysmetria	CNS (brain MRI)	143 cells/μl, VL < 50 copies/ml	didanosine, lamivudine, indinavir	i.v.: ciprofloxacin 400 mg tid vancomycin 500 mg q6h ceftriaxone 2 g qd	3 weeks

Notes. *VL *viral load; *i.v. *intravenous; *p.o. *per os; *CNS *central nervous system; *MRI *magnetic resonance imaging

At the time of admission in April, CD4+ cell count was 123/μL and HIV-RNA was undetectable. Chest X-ray showed a nodular opacity in the left upper lobe and *R. equi *was cultured in blood and sputum, leading to a diagnosis of *R.equi *pneumonia. Intravenous treatment with vancomycin, imipenem, ceftriaxone and ciprofloxacin was started, along with oral prednisone (75 mg qd for 8 days, then tapered in 20 days) for a concomitant, likely HIV-related, severe thrombocytopenia. In May 2002, the patient presented with generalized seizures. Brain magnetic resonance imaging (MRI) confirmed the presence of the known vasculitis-like lesions, still contrast enhancing, but also showed an extracranial abscess, which was surgically removed and from which *R. equi *was cultured. Intravenous antibiotic treatment was continued for a total of 8 weeks, then the patient received oral ciprofloxacin and chlarithromycin for the following 15 weeks. In July 2002 patient's respiratory symptoms resolved, CD4+ cells count was 161/μL and HIV RNA 10,800 c/ml (patient had discontinued therapy in May because of diarrhoea and resumed same HAART in June), chest X-ray and CT scan showed complete regression of the lung nodules. Because of new onset of cognitive symptoms, and persistence of the contrast-enhancing vasculitis-like brain lesions, oral prednisone was started at 1 mg/Kg and gradually tapered for a total of five months.

**Figure 1:**
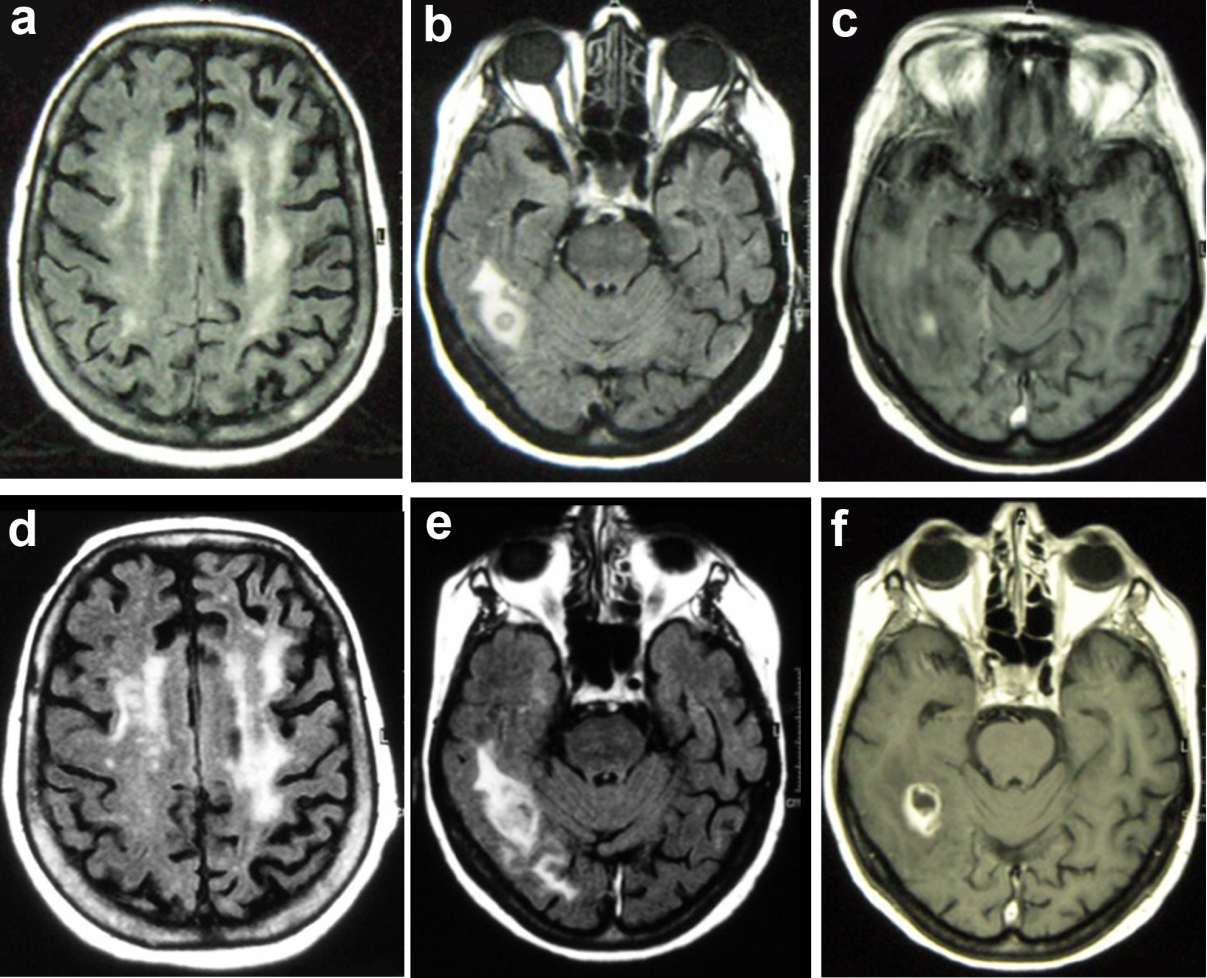
**Central nervous system (CNS) immune reconstitution white matter lesions and *R. equi *brain abscesses by Magnetic Resonance Imaging (MRI) (case report 1)**. **a-c**. Brain lesions at the onset of focal neurological symptoms. **a**. Axial FLAIR brain sequence showing non specific asymmetric bilateral hyperintensity in the subcortical region, expression of immune recconstitution inflammatory reaction. **b**. Axial FLAIR sequence showing abscessual lesion, surrounded by oedema, in the right temporal region. **c**. Gadolinium (Gd)-T1 sequence shows the presence of nodular enhancement of the right temporal lesion. **d-f**. Evolution of brain lesions 1 month after onset of symptoms. **d**. Axial FLAIR sequence shows the persistence of the non specific white matter hyperintensity; **e**. Axial FLAIR sequence shows an increase of lesion size and oedema of the right temporal lesion; **f**. Gd-T1 sequence shows the evolution of contrast enhancement, now presenting as ring enhancement, typical of an abscessual lesion.

In December 2002, about 20 weeks after interruption of anti-rhodococcal treatment, patient developed anisocoria, right hemiparesis, hypoaesthesia, hypopallaesthesia, and dysmetria. At MRI, the known white matter lesions were still present but no longer contrast enhancing (Figure 1a), however, the exam showed two new contrast-enhancing nodular lesions, surrounded by oedema (Figure 1b, c). Cerebrospinal fluid (CSF) examination was unremarkable, no bacteria, mycobacteria and fungi were cultured, cryptococcal antigen and Epstein-Barr virus DNA were undetectable. Antitoxoplasmic treatment was given empirically for 3 weeks, without improvement of clinical conditions, and followed by enlargement of the nodular lesions at MRI (Figure 1d-f). In the suspect of brain rhodococcosis, intravenous treatment with ciprofloxacin, vancomycin and ceftriaxone was given for 3 weeks, together with oral prednisone, followed by resolution of focal symptoms and improvement of MRI lesions. During the following years, severe neurological impairment with gait impairment persisted, the patient developed progressive dementia and was admitted to a long-term medical facility. Follow-up brain MRIs documented the disappearance of the abscesses, persistence of the vasculitis-like lesions and development of cerebral atrophy. Patient changed HAART for toxicity many times, and her immunovirological situation remained stable. She did not develop rhodococcosis relapses or other opportunistic complications, but died in December 2010 for ischemic cardiac events.

### Case report 2

**Figure 2:**
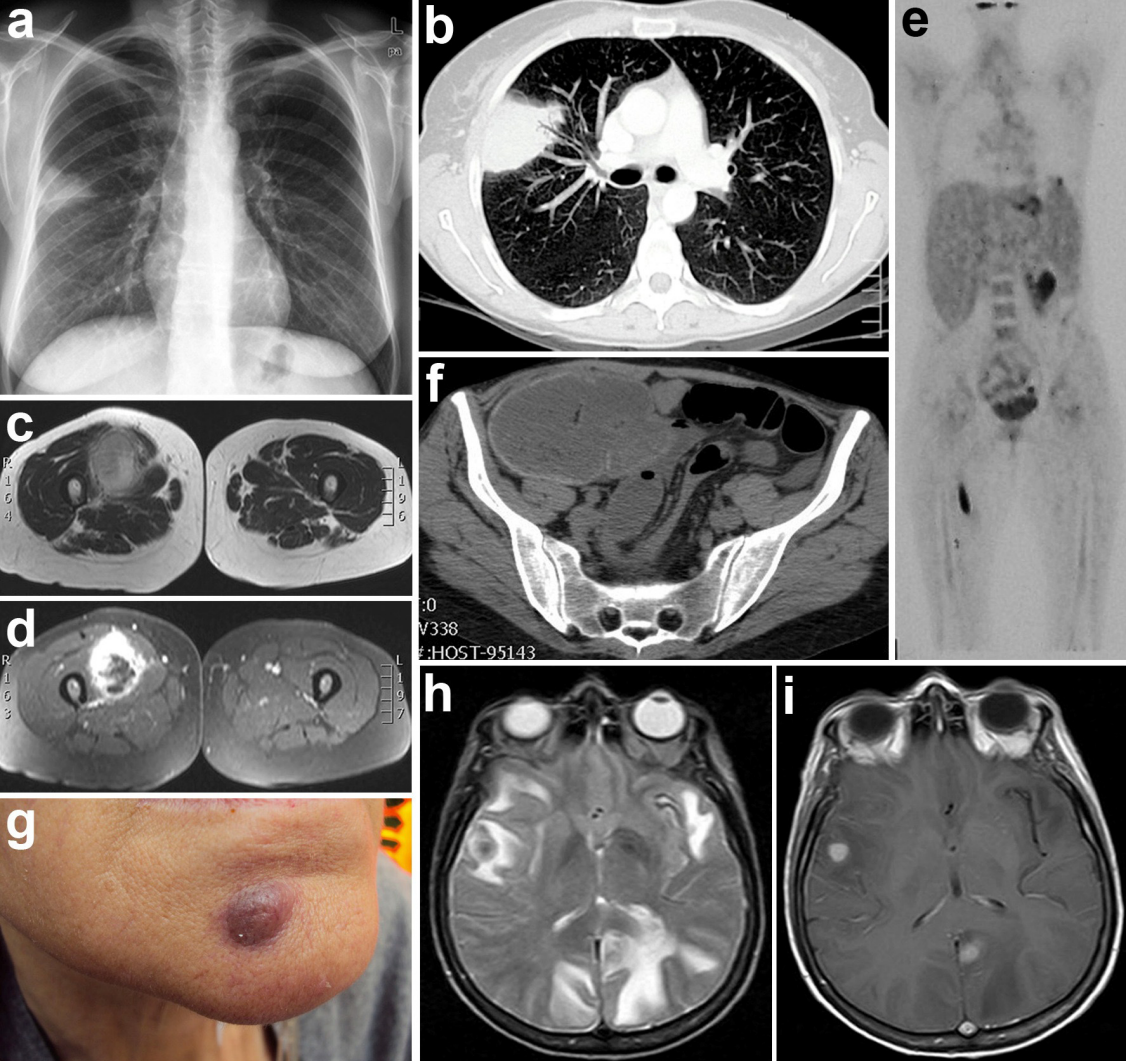
***R. equi *lesions in disseminated infection (case report 2)**. **a **Chest radiograph (May 2005) shows a non specific subpleural opacity in the right upper lobe without evidence of pleural effusion. **b **Contrast enhanced multidetector computed tomography (MDCT)(May 2005). A scan at the level of the main bronchi demonstrates a subpleural focal consolidation in the right upper lobe. There is no evidence of lymphoadenopathy or pleural effusion. **c**, **d **Contrast enhanced magnetic resonance imaging (MRI) of the right thigh (August 2009). The axial T2 sequence (**c**) and the axial T1 contrast enhanced sequence with fat suppression (ccGRE T1FS) (**d**) show an oval shaped enhancing mass in the vastus medialis muscle with central area of necrosis and oedema of the surrounding tissue and muscle. **e **Positron emission tomography (PET) and computed tomography (CT) images (August 2009) show focal increased fluodeoxyglucose (FDG) uptake in the upper lobe of the right lung and in the spleen, which is larger than normal, a large area of increased uptake in the right lower abdomen consistent with colon localization, and an irregular area of increased FDG uptake in the soft tissue of the right proximal thigh. **f **MDCT of the abdomen (March 2010). A scan through the lower abdomen shows a large obstructing mass in the right colon with stranding of the pericolonic fat and several enlarged lymph nodes. **g **Cutaneous nodular rhodococcal lesions (March 2010). **h **Brain axial T2 weighted sequence (August 2010) shows multiple (right temporal, left mesial occipital, left temporoinsular) expansive oedematous lesions. All the lesions show central hypointensity and peripheral hyperintensity. Oedema is also present in right occipital and anterior temporal lobes. **i **Brain axial T1 weighted sequence after Gd injection (August 2010) shows enhancement of the two nodular lesions in right temporal region and in left occipitomesial lobe. Smooth cortical enhancement is also seen in the left occipital lobe.

A 45 year old, HIV-infected woman, with a history of breast carcinoma successfully treated with quadrantectomy and local radiotherapy, presented in May 2005 with low-grade fever (below 38°C), cough and dyspnea (Table 2). She was off HAART since 2000, her CD4+ cells count was 133/μl and HIV RNA 40,700 c/ml. She was on cotrimoxazole as primary *P. jiroveci *pneumonia prophylaxis. Chest X-ray and CT scan showed a 5 cm pulmonary nodule in the upper right pulmonary lobe (Figure 2a, b). *R. equi *was isolated from expectorate and, based on the results of antibiogram, patient received 2 weeks of intravenous rifampicin, levofloxacin and azythromicin, followed by 8 weeks of oral levofloxacin and azythromicin. HAART with lamivudine, tenofovir and efavirenz was also started. Respiratory symptoms resolved with progressive reduction of the pulmonary lesions. In August, CD4+ cells count was 60/μl and HIV RNA was undetectable (<50 c/ml). During the following years, patient adherence to HAART remained poor until she stopped treatment in 2008. Her CD4+ cells count always remained below 200/μl, nevertheless, she neither experience respiratory diseases nor any other HIV-related manifestations.

**Table 2:** Summary of main clinical, laboratory and imaging findings from patient 2

Onset of symptoms (date)	Presenting symptoms	Site of R. equi infection (diagnostic technique)	CD 4 + cells, HIV-1 RNA (VL)	HAART	Treatment	Duration
May 2005	fever, cough, dyspnea	lung (culture of sputum, chest X-ray, chest high resolution CT scan)	133 cells/μl, VL: 40,700 copies/ml	lamivudine, tenofovir, efavirenz	induction (i.v.): rifampicin 600 mg qd, levofloxacin 500 mg qd, azythromicin 500 mg qd maintenance (p.o.): levofloxacin 500 mg qd azytromicin 500 mg qd	induction: 2 weeks maintenance: 8 weeks

September 2009	weight loss, fever, subcutaneous nodule in the right thigh	subcutaneous thigh tissue (culture of granuloma, MRI, needle aspirate); colon (colonoscopy with biopsy)	90 cells/μl VL: 78,650 copies/ml	emtricitabine, tenofovir, atazanavir	azythromicin i.v. 500 mg qd, levofloxacin i.v. 750 mg qd, ryfampicin i.v. 600 mg qd, switched to rifabutin p.o. 150 mg q48h at beginning of HAART	8 weeks

November 2009	fever, ascites, diarrhoea	colon, peritoneum, abdominal lymph nodes, lung (total body CT and PET scan)	59 cells/μl, VL: 778 copies/ml	emtricitabine, tenofovir, darunavir/r	induction (i.v.): imipenem 1 g q6h amikacin 1 g qd maintenance (p.o.): azythromicin 500 mg qd, levofloxacin 750 mg qd	induction: 3 weeks maintenance: 10 weeks

March 2010	fever, abdominal pain and intestinal bleeding	disseminated infection (blood culture); colon (CT scan and histological examination of surgically removed colonic tissue); lung (biopsy); subcutaneous thigh tissue (culture of needle aspirate, MRI); skin.	62 cells/μl VL < 50 copies/ml	emtricitabine, tenofovir, darunavir/r	surgery induction (i.v.): imipenem 500 mg q6h, switched to ertapenem 1 g qd after 7 weeks, levofloxacin 750 mg qd, vancomicin 500 mg bid, azythromicin 500 mg qd maintenance (p.o.): azythromicin 500 mg qd, rifabutin 150 mg q48h	induction: 14 weeks maintenance: 8 weeks

August 2010	headache	CNS (brain CT scan and MRI)	74 cells/μl VL < 50 copies/ml	emtricitabine, tenofovir, darunavir/r	induction (i.v.): imipenem 500 mg q6h, amikacin 1 g qd maintenance (i.v.): meropenem 1 g q8h, azythromicin 500 mg qd, switched to ertapenem 1 g qd after 10 weeks	induction: 3 weeks maintenance: 10 weeks

Notes. *VL *viral load; *i.v. *intravenous; *p.o. *per os; *CNS *central nervous system; *MRI *magnetic resonance imaging

In the summer of 2009, 4 years after *R. equi *pneumonia, patient reported a weight loss of 10 Kg in 3 months and low-grade fever, and noticed a subcutaneous, non painful nodule in the right thigh. CD4+ cells count was 90/μL and HIV RNA 78,650 c/mL. An MRI of the tight lesion was highly suggestive for a neoplastic lesion, with necrosis and inflammation (Figure 2c, d), which prompted a diagnostic work-up for tumor identification and staging. A CT scan showed multiple abdominal enlarged lymph nodes, and a 18-fluodeoxyglucose (FDG) PET/CT scan showed increased metabolic activity of the thigh lesion, colon and spleen (Figure 2e). However, a needle biopsy of the thigh nodule demonstrated a non necrotic granuloma, from which *R.equi *was cultured. A colonoscopy showed a polipoid, stenotic lesion, which histologically disclosed a picture of malakoplakia, with the presence of microorganisms with morphology and staining features consistent with *R.equi*. Based on the antibiogram, azythromicin, levofloxacin and ryfampicin was started in September 2009. HAART with tenofovir, emtricitabine and atazanavir was introduced 1 month later, with rifampicin replaced by rifabutin, followed by prompt virological response, but no significant CD4+ cells increase.

During the following weeks, patient's conditions continued to worsen, with further weight loss, fever persistently above 38°C, and onset of dyspnea, ascites and diarrhoea. An abdominal MRI showed a 4 cm wide lesion of colon and a 3 cm wide lesion in perisplenic peritoneum; abdominal, celiac, para-hepatic and para-aortic enlarged lymph nodes of 1-2 cm of diameter, peritoneal and pleural effusion, while a chest high resolution CT scan showed multiple lung consolidations. In November, antibiotic treatment was switched to imipenem and amikacin, whilw HAART was modified with atazanavir replaced by darunavir/ritonavir, followed by significant clinical improvement and reduction of both dimension and metabolic activity of the thigh lesion and abdominal lymph nodes, as documented by conventional CT and 18-FDG PET/CT scans. After 3 weeks, treatment was replaced by oral levofloxacin and azythromicin. Remarkably, a new gastric hypercaptation was noted at 18-FDG PET/CT scan. In January 2010, the patient received extracorporeal lithotripsy for nephrolithiasis, possibly consequent to granulomatosis-associated hypercalciuria. At this time there was no longer evidence of the thigh lesion.

In February 2010, after 10 weeks of anti-rhodococcal oral therapy, patient self-suspended antibiotic and antiretroviral therapy for severe gastralgia. An esophago-gastro-duodenoscopy (EGDS) showed esophageal candidiasis and the presence of a large stomach ulceration, which was histologically proved to be a large B cell diffuse lymphoma. CD4+ cells count was 62/μL and HIV-RNA undetectable (<50 c/mL). Patient started proton pump inhibitors and fluconazole.

In March 2010 the patient was admitted to Hospital because of abdominal pain and intestinal bleeding requiring blood transfusion. CT scan showed a solid lesion in the right colon (Figure 2f), surrounded by multiple enlarged lymph nodes, with a radiologic appearance of a colon cancer; colonscopy showed a large mass in the proximal ascending colon, which was biopsied. However, in the following days the patient developed peritonitis and caecal perforation that required emergency laparotomy and right hemicolectomy. Histological examination of a 7 cm wide sessile, centrally excavated, heteroplasia in the ascending colon and of peritoneal lymphonodes showed malakoplakia of the intestine and of perivisceral lymph nodes [23]. A similar histological picture was disclosed by an echo-guided biopsy of a 2.5 cm wide lesion of the chest wall. Patient noticed also the appearance of subcutaneous nodules on her face (Figure 2g) and reappearance of the thigh lesion and *R. equi *was isolated from both the thigh lesion and blood samples. Based on antibiogram, patient was started again on imipenem, levofloxacin and vancomicin; intravenous azythromicin was added after 3 weeks. Vancomicin was stopped after 4 weeks for severe thrombocytopenia, imipenem replaced by ertapenem after 7 weeks and levofloxacin suspended after 8 weeks. After a total of 14 weeks, intravenous treatment was substituted with oral azythromicin and rifabutin. In June 2010, patient's conditions were improved, with disappearance of the skin lesions, volume reduction of the thigh lesion and lymph nodes at CT scans and no abnormalities in lungs and brain. Multiple biopsies on a control EGDS did not confirm the presence of gastric lymphoma. Despite virological suppression, there was no immunological improvement (74 CD4+ cells count/μL).

In August 2010, patient was admitted to hospital for important headache, CT and MRI disclosed multiple brain contrast-enhancing lesions, associated with cerebral oedema (Figure 2h, i). Intravenous imipenem and amykacin were started together with mannitol and dexamethasone, followed by clinical improvement. Systemic spread of rhodococcosis was excluded by a total body CT scan. After 21 days treatment was changed to meropenem and azythromicin and, after 10 weeks, to ertapenem alone. MRI follow-up showed progressive reduction of cerebral lesions, despite occurrence of seizures in December 2010, for which antiepileptic therapy was started.

## Discussion and conclusions

Highly active antiretroviral therapy has impressively reduced mortality and morbidity of opportunistic diseases [24]. Nevertheless, these remain significant in patients with low CD4+ cells count and during the first months of therapy. This may either result from insufficient immunological recovery, or from the inflammatory reaction to the opportunistic infection - the so called immune reconstitution inflammatory syndrome (IRIS)- [25,26].

None of the patients here described was indeed able to increase the CD4+ cells count during the first months of HAART, despite complete virological suppression, and this was associated with the multi-organ spread of rhodococcosis. Lack of immunological recovery, indeed, is associated with enhanced risk of disease progression and death, even in virologically suppressed patients [27]. Conversely, in the first patient, the immunological recovery after CNS relapse was accompanied by a long disease-free period.

The integrity of cellular immunity appears essential for clearing *R. equi *infection [22]. During infection, *R. equi *survives inside macrophages by inhibiting the formation of the phagosome-lysosome and thereby its degradation [28]. In vitro production of IFN-γ and TNF-α in response to stimulation with *R. equi *is significantly impaired in AIDS patients compared to healthy subjects [29], confirming the importance of adequate cellular, and especially Th1 response for clearing the infection. It is possible that the persistence of *R. equi *in macrophages also impairs the function of these cells, thus contributing to maintain the immunodeficiency. Indeed, lack of CD4+ T cell rise despite full virological suppression has also been observed in other HIV-associated opportunistic infections, such as visceral leishmaniasis [30], also characterized by the persistence of the parasites in macrophages [31]

In our first case, rhodococcal pneumonia occurred 6 months after the introduction of HAART, right after a manifestation of CNS-IRIS, suggesting an immune reconstitution associated event. Indeed, this case met the definition criteria for "unmasking" IRIS, i.e., unmasking or paradoxical deterioration of an opportunistic disease after introduction of an effective antiretroviral therapy [26,32]. A case of paradoxical worsening of *R. equi *pneumonia following HAART initiation was also recently described (i.e., paradoxycal IRIS)[33]. These observations suggest that *R. equi *might be added to the list of pathogens associated with IRIS. As for other opportunistic infections, the first weeks after introduction of HAART are critical to this regard and patients should be strictly monitored in order to early recognize IRIS events. However, experience is still limited to determine best timing of HAART introduction in the context of rhodococcosis or use of antinflammatory drugs if IRIS develops.

One important clinical issue in our second case was the difficult diagnostic approach, which required invasive techniques, such as needle biopsy and endoscopic biopsy because of the difficult interpretation of the radiological findings. In this setting the potential of "non-conventional" techniques in the diagnostic work-up was remarkable, with 18-FDG PET/CT that proved useful to uncover silent localizations of the disease, such as the colic and peritoneal lesions. This technique is a sensitive tool not only in neoplastic diseases, but also in tuberculosis and invasive aspergillosis [34-36]. It was also reported to be useful to disclose a rhodococcal localization of the tongue [37], but it is not an established means for diagnosis or monitoring of rhodococcosis. Like in tuberculosis, high 18-FDG uptake in rhodococcal lesions is likely related to high level of cell proliferation, possibly in relation to the inflammatory process. It was challenging, in our second patient, to recognize *R.equi *as the responsible of lesions, such as those at thigh, colon and lung, the radiological appearance of which resembled that of a neoplastic lesion, in a patient with previous history of neoplasia and histological evidence of gastric lymphoma.

While it is difficult to dissect the effect of individual anti-rhodococcal agents in disseminated infection, it appeared that a regimen containing carbapenems was more effective in our second case than a combination of rifampicin, levofloxacin and azythromicin, which had previously been effective to cure pulmonary infection. On the other hand, a combination of imipenem and aminoglicosides, given for 3 weeks and followed by antibiotic maintenance therapy, although partially effective, did not prevent subsequent progression of extrapulmonary infection. Similarly, the subsequent 14 week course with carbapenems, followed by oral antibiotic maintenance, did not prevent later occurrence of CNS lesions. In this regard, it is of note the dissemination of the infection to the CNS in both of our cases, which occurred in the first case months after apparent recovery of *R.equi *pneumonia and, in the second case, despite long-term induction and current maintenance therapy. CNS involvement could have been due to spread of infection to the CNS at the time of extra-cerebral disease, kept under control by systemic treatment, and reactivated once antimicrobial treatment was interrupted, such as in the first case, or switched to an oral maintenance regimen, such as in the second case.

The cases here presented show that rhodococcosis may disseminate to a number of tissues in HAART-treated immunological failing patients, and indicate that long-term intravenous treatment might be required to avoid relapses at distance, such in CNS, at least until a sufficiently high CD4+ cell count, e.g., more than 200/μL, is achieved. Achieving complete immune reconstitution by means of HAART remains the most important weapon against rhodococcosis, in addition to combination antimicrobial treatment.

## Competing interests

The authors declare that they have no competing interests.

## Authors' contributions

All the authors contributed to the care and diagnosis of the patients. FF drew up the first draft of the report, PC made a substantial contribution to draft the manuscript and revised the draft. All authors read and approved the final version of the manuscript.

## Consent

Written informed consent was obtained from patients for publication of this study. A copy of the written consent is available for review by the Editor-in-Chief of this journal.

## Pre-publication history

The pre-publication history for this paper can be accessed here:

http://www.biomedcentral.com/1471-2334/11/343/prepub
